# Validation of a Gas Chromatography-Mass Spectrometry Method for the Measurement of the Redox State Metabolic Ratios Lactate/Pyruvate and β-Hydroxybutyrate/Acetoacetate in Biological Samples

**DOI:** 10.3390/ijms22094752

**Published:** 2021-04-30

**Authors:** Robin Wijngaard, Meritxell Perramón, Marina Parra-Robert, Susana Hidalgo, Gina Butrico, Manuel Morales-Ruiz, Muling Zeng, Eudald Casals, Wladimiro Jiménez, Guillermo Fernández-Varo, Gerald I. Shulman, Gary W. Cline, Gregori Casals

**Affiliations:** 1 Service of Biochemistry and Molecular Genetics, Hospital Clinic Universitari, Centro de Investigación Biomédica en Red de Enfermedades Hepáticas y Digestivas (CIBERehd), Institut d’Investigacions Biomèdiques August Pi i Sunyer (IDIBAPS), Carrer de Villarroel 170, 08036 Barcelona, Spain; wijngaard@clinic.cat (R.W.); mperramon@clinic.cat (M.P.); mparra@clinic.cat (M.P.-R.); shidalg1@clinic.cat (S.H.); morales@clinic.cat (M.M.-R.); wjimenez@clinic.cat (W.J.); 2 Department of Internal Medicine, Yale School of Medicine, New Haven, CT 06510, USA; ginabutrico@gmail.com (G.B.); gerald.shulman@yale.edu (G.I.S.); gary.cline@yale.edu (G.W.C.); 3 Department of Biomedicine, University of Barcelona, 08036 Barcelona, Spain; 4 Working Group for the Biochemical Assessment of Hepatic Disease-SEQCML, 08036 Barcelona, Spain; 5 School of Biotechnology and Health Sciences, Wuyi University, 99 Yingbing Middle Rd., Jiangmen 529020, China; mulingzeng@163.com (M.Z.); eudaldcm@gmail.com (E.C.)

**Keywords:** redox state, GC-MS, microwave-assisted derivatization, nicotinamide adenine dinucleotide, ketone bodies

## Abstract

The metabolic ratios lactate/pyruvate and β-hydroxybutyrate/acetoacetate are considered valuable tools to evaluate the in vivo redox cellular state by estimating the free NAD+/NADH in cytoplasm and mitochondria, respectively. The aim of the current study was to validate a gas-chromatography mass spectrometry method for simultaneous determination of the four metabolites in plasma and liver tissue. The procedure included an o-phenylenediamine microwave-assisted derivatization, followed by liquid-liquid extraction with ethyl acetate and silylation with bis(trimethylsilyl)trifluoroacetamide:trimethylchlorosilane 99:1. The calibration curves presented acceptable linearity, with a limit of quantification of 0.001 mM for pyruvate, β-hydroxybutyrate and acetoacetate and of 0.01 mM for lactate. The intra-day and inter-day accuracy and precision were within the European Medicines Agency’s Guideline specifications. No significant differences were observed in the slope coefficient of three-point standard metabolite-spiked curves in plasma or liver and water, and acceptable recoveries were obtained in the metabolite-spiked samples. Applicability of the method was tested in precision-cut liver rat slices and also in HepG2 cells incubated under different experimental conditions challenging the redox state. In conclusion, the validated method presented good sensitivity, specificity and reproducibility in the quantification of lactate/pyruvate and β-hydroxybutyrate/acetate metabolites and may be useful in the evaluation of in vivo redox states.

## 1. Introduction

Metabolic ratios lactate/pyruvate and β-hydroxybutyrate/acetoacetate are widely used to assess redox states in different experimental [1,2,3,4] and clinical scenarios [5,6,7,8,9]. As initially described by Williamson et al. [10], free NAD^+^/NADH in the cytoplasm can be calculated using the lactate/pyruvate ratio whereas free NAD^+^/NADH in the mitochondria can be calculated using the β-hydroxybutyrate/acetoacetate ratio. Although it is the free form of NAD^+^ and NADH which regulates cellular redox potential, current techniques can only reliably measure total NAD^+^/NADH and thus could not differentiate between the more abundant protein-bound form and the free (active) form [11,12]. In addition, the free NAD^+^/NADH ratios of cytoplasm and mitochondria are different and do not necessarily move in parallel when the cell metabolic state changes [13]. Thus, advantages of measuring metabolic ratios include overcoming the difficulties associated with free NAD^+^/NADH measurements and the ability to calculate separated redox states in the cytoplasm and mitochondria.

The four metabolites composing the metabolic ratios can be measured using different methods including enzymatic assays [14,15,16,17] and mass spectrometry-based methods [18,19,20,21]. However, the measurement of these metabolites has some difficulties owing to their instability, especially in the case of pyruvate and acetoacetate, and to the low concentrations in some type of samples [22,23]. Mass spectrometry methods present some advantages over other methods since they are very sensitive and highly specific.

In the current study, we present a gas chromatography-mass spectrometry (GC-MS) method specifically devised and validated for the determination of plasma and liver tissue levels of lactate/pyruvate and β-hydroxybutyrate/acetoacetate ratios. The intended use is the measurement using the same procedure of both ratios and the four metabolites in the clinical and research settings.

## 2. Results and Discussion

### 2.1. Method Optimization

The aim of the present work was to develop a simple, sensitive and reproducible method to allow the accurate quantification of both lactate/pyruvate and β-hydroxybutyrate/acetoacetate ratios in plasma and tissue which may be useful in the evaluation of in vivo redox states. Here, we present a GC-MS method that allows both metabolic ratios to be measured in the same aliquot of plasma or tissue sample. Liquid-liquid extraction was conducted for its simplicity and a high signal and cleanliness were obtained using ethyl acetate. The initial tests revealed the need for previous acidification of samples to achieve appropriate sensitivity. In addition, metabolites should be derivatized to improve detectability and the efficiency of the GC. Derivatization with a mixture of bis(trimethylsilyl)trifluoroacetamide (BSTFA) with 1% trimethylchlorosilane (TMCS) after acidic ethyl acetate extraction was initially tested for its readiness. Lactate, β-hydroxybutyrate and acetoacetate were successfully detected. Conversely, pyruvate proved to be difficult to detect. Sylilation with *N*-trimethylsilylimidazole with or without the addition of methoxyamine hydrochloride also did not achieve pyruvate derivatization. Finally, the obtaining of quinoxalinol-trimethylsilyl (TMS) derivative was tested. Excellent sensitivity and chromatographic detection of pyruvate were offered by obtaining quinoxalinol derivative using o-phenylenediamine (in 4 M HCl) at a 1:2 volume ratio with the sample before extraction and silylation with BSTFA with 1% TMCS.

**Figure 1:**
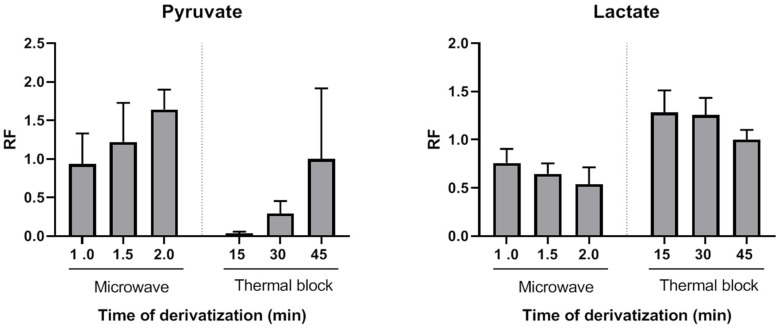
Comparison of derivatization yields of pyruvate and lactate after quinoxalinol deritvatitzation step performed using microwave or thermal block at different incubation times (mean of three plasma pools). RF: response factor.

Quinoxalinol-TMS derivatization has been previously used for 2-ketoacids detection using GC [24], GC-MS [21] and HPLC-fluorescence [25] with derivatization times ranging from 20 min to 90 min. Here, we evaluated the usefulness of a MAD approach to obtain fast and stable pyruvate quinoxalinol derivatives. Microwave-assisted derivatization (MAD) of pyruvate was initially tested with aqueous solutions of pyruvate standard (0.1 µmol). A successful derivatization was achieved after 1 min of microwave irradiation of the standard mixed with o-phenylenediamine. Peak areas after 1.5 or 2 min of microwave irradiation were higher than those obtained after incubation at 90 °C for 45 min. To further investigate the applicability of MAD of pyruvate in plasma samples, this alternative heating approach was compared with the classical block heating method also in plasma samples (*n* = 3). Aliquots of the same samples were analyzed using both methods and derivatization yields for pyruvate were compared. Figure 1 shows the average relative response factors (RRFs) values of MAD normalized to classical derivatization (45 min, 90 °C). The MAD derivatization method produced similar or higher absolute yields. RRFs ranged from 0.94 (1 min MAD) to 1.64 (2 min MAD). Thus, by using the described MAD procedure, the quinoxalinol derivatization step can be reduced to only a very few minutes of microwave irradiation. To the best of our knowledge, this is the first report of MAD quinoxalinol derivatization of pyruvate. Although only pyruvate derivatization yields were evaluated, results suggest that this MAD procedure may be useful for the analysis of other 2-ketoacids or methods aiming at comprehensive acids organics profiling.

The effect of pyruvate quinoxalinol derivatization on the yields of lactate, β-hydroxybutyrate and acetoacetate TMS derivatives was also evaluated. As shown in Figure 1, both thermal and MAD derivatization reactions of pyruvate with o-phenylenediamine resulted in time-dependent lower yields of lactate. This decrease was probably due to a temperature effect, as it was more pronounced in the case of microwave irradiation. However, a synergic effect of temperature and o-phenylenediamine cannot be excluded. The average RRFs values of MAD normalized to classical derivatization (45 min, 90 °C) were lower and ranged from 0.76 (1 min MAD) to 0.54 (2 min MAD). However, as it will be shown, this did not preclude achieving high sensitivity, precision and accuracy for lactate measurements. β-hydroxybutyrate decrease of RRFs after incubation with o-phenylenediame via thermal block or MAD were similar to those observed for lactate, but acetoacetate was not detected after the thermal or MAD quinoxalinol derivatization step. Therefore, this step was only applied to the measurement of the first ratio (lactate/pyruvate) and avoided for the measurement of the second ratio (β-hydroxybutyrate/acetoacetate) by splitting the sample into two extracts (tube 1 and tube 2) and handling them in parallel in the remaining part of the assay procedure. It is noteworthy that lactate and β-hydroxybutyrate were correctly detected in both tubes 1 and 2. However, the metabolites were analyzed in pairs in order to keep the procedure as homogeneous as possible between the metabolites that constitute each ratio.

Extraction with ethyl acetate required acidic conditions, which were provided by adding a solution of o-phenylenediamine prepared in 4 M hydrochloric acid (HCl) or by directly adding a 4 M HCl solution. Without acidification, none of the metabolites were detected, except for lactate, the peak area of which was reduced by 99.3%. We did not find differences between the yields of the metabolites using HCl concentrations of 4 M, 5 M, 7.5 M or 10 M, whereby 4 M HCl was chosen. After extraction with ethyl acetate in acidic conditions, metabolites were silylated with BSTFA with 1% TMCS using MAD (1 min irradiation) as previously described [26,27]. In this way, TMS derivatives of lactate, β-hydroxybutyrate and acetoacetate were obtained, and the approach also proved to be successful for the derivatization of quinoxalinol pyruvate.

**Figure 2:**
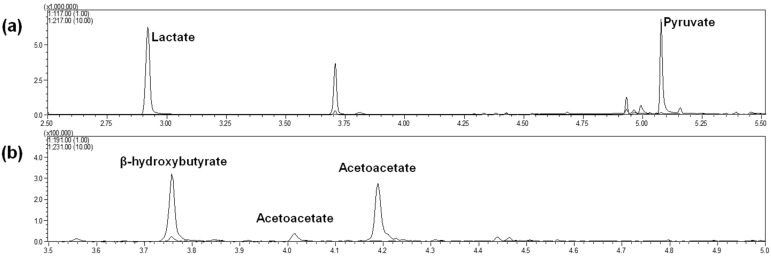
(**a**) Mass chromatograms of a rat liver sample for lactate (*m*/*z* 117) and pyruvate (*m*/*z* 217) (tube 1). The concentrations were 1.23 mM and 0.18 mM, for lactate and pyruvate, respectively. Note that an additional peak can be observed at 3.7 min, derived from the elution of β-hydroxybutyrate, which was stable against the MAD quinoxalinol derivatization step. (**b**) Mass chromatograms of a rat liver sample for β-hydroxybutyrate (*m*/*z* 191) and acetoacetate (*m*/*z* 231) (tube 2). The concentrations were 0.56 mM and 0.031 mM, for β-hydroxybutyrate and acetoacetate, respectively.

Two separate injections were finally performed for each initial sample: one for lactate and pyruvate (with the MAD quinoxalinol derivatization step) and one for β-hydroxybutyrate and acetoacetate (without the MAD quinoxalinol derivatization step). Chromatographic conditions led to the profiles shown in Figure 2a for lactate and pyruvate (injection of tube 1) and Figure 2b for β-hydroxybutyrate and acetoacetate (injection of tube 2). Metabolites were completely separated via GC. Lactate and pyruvate were eluted after 2.9 min and 5.1 min, respectively, whereas β-hydroxybutyrate, acetoacetate (peak 1) and acetoacetate (peak 2) were eluted after 3.7, 4.0 and 4.2 min, respectively. Note that an additional peak can be observed in Figure 2a at 3.7 min, derived from the elution of β-hydroxybutyrate, which was stable against the MAD quinoxalinol derivatization step. The appearance of two peaks for acetoacetate, with identical mass spectra, has been previously noted [19,28] and is probably related to the formation of tautomeric isomers. Only the peak with the greater abundance, eluted at 4.2 min, was selected for the method validation since the minor peak at 4.0 min was often too small for appropriate peak integration and no improvement in the validation results was observed when using both peaks. Finally, synchronous SIM/scan acquisition was used to achieve low detection limits while maximizing specificity. The selected ion values and retention times of metabolite derivatives are given in Table 1, and the mass spectra are shown in Figure S1.

To the best of our knowledge, only one previous study validated a method for the simultaneous measurement of the four metabolites using mass spectrometry [18]. In this study [18] the same amount of plasma (200 µL) was used, and ethyl acetate was also used for extraction. However, methanol was used for protein precipitation, and a derivatization with N-methyl-N-(trimethylsilyl)trifluoroacetamide (MSTBFA) was performed before GC-MS analysis. Furthermore, in contrast with our study, tissue samples were not included in the validation. In a previous study, Beylot et al. [19] used a GC-MS method to measure the isotopic enrichment of acetoacetate and β-hydroxybutyrate. In contrast to our method, 2 mL of whole blood were used, and protein precipitation was performed with perchloric acid. However, samples were similarly acidified with HCl, extracted with ethyl acetate and derivatizatied with BSTFA with 1% TMCS, although lactate and pyruvate measurements were not performed. Finally, the derivatization of pyruvate along with other 2-ketoacids using o-phenyldiamine was previously described by Rocchiccioli et al. [21]. The method was applied to measure pyruvate in blood, plasma and urine. After reaction with o-phenyldiamine (1 h, 90 °C) samples were extracted twice with ethyl acetate and derivatized with BSTFA-TMCS (30 min, 90 °C). In our method, the use of MAD for the same reactions resulted in considerably shorter derivatization times.

### 2.2. Method Validation

Calibration curves were prepared in water to avoid the potential bias resulting from endogenous metabolites at different concentrations in plasma and tissue. The method was linear for lactate concentrations ranging from 0.01 to 5 mM, for pyruvate and acetoacetate concentrations ranging from 0.001 to 1 mM and for β-hydroxybutyrate ranging from 0.001 to 5 mM, obtaining r^2^ values of 0.999 ± 0.001, 0.998 ± 0.001, 0.998 ± 0.003 and 0.998 ± 0.001, respectively. The concentrations of the calibration curves were established taking into account the expected variation of the metabolite concentrations in different types of samples. These concentrations were also estimated via initial injection of plasma and liver samples. Therefore, a wide range of standard concentrations was selected since metabolite concentrations may change widely in different pathological and experimental conditions. In spite of this, calibration samples showed an accuracy above 85% and a precision below 15% (Table 2), as specified in the current guidelines for analysis [29]. The low calibrator of 0.001 mM (pyruvate, β-hydroxybutyrate and acetoacetate) or 0.01 mM (lactate) was chosen for the low limit of quantification (LloQ). The inter-day accuracy and precision of the LloQ were within 100 ± 20% and below 20%, respectively, except for acetoacetate LloQ precision, which was 22% (Table 2).

Although isotopic internal standards were used to compensate for any variations during sample processing, additional validation procedures were necessary to evaluate the appropriateness of preparation of calibrations in water. Experiments were performed to evaluate the differences in recoveries between plasma or liver and water. Table S1 shows the slope coefficients of 3-point QC metabolite-spiked curves in plasma, liver or water. Acceptable recoveries of added metabolites were obtained when analyzing spiked plasmas and livers (Table 3) supporting the parsimonious approach of not compensating for the different matrices.

Table 4 summarizes the values of intra-day and inter-day precision and accuracies obtained for the three QC levels, which were <15%. In addition, intra-day and inter-day precision for human plasma and rat liver samples were evaluated and shown to be <15% with the exception of an inter-day precision value of 22% for acetoacetate in the liver (Table 5). The calculated lactate/pyruvate and β-hydroxybutyrate/acetoacetate ratios in these samples were in the range of 10.5–12.4 and 5.7–5.8 in plasma and 6.9–18.4 and 16.0–19.3 in the liver, respectively.

No interfering signals were observed in any metabolite when analyzing 10 different human plasma samples and 3 different rat livers. In addition, there were no carry-over effects after injecting blank samples (1 µL of cyclohexane) following an injection of a standard with UloQ concentration. Finally, extracts in the autosampler at ambient temperature were highly stable for at least 96 h (Table 6). This result further supports the efficacy of MAD quinoxalinol derivatization of pyruvate.

### 2.3. Applicability of the Method

**Figure 3:**
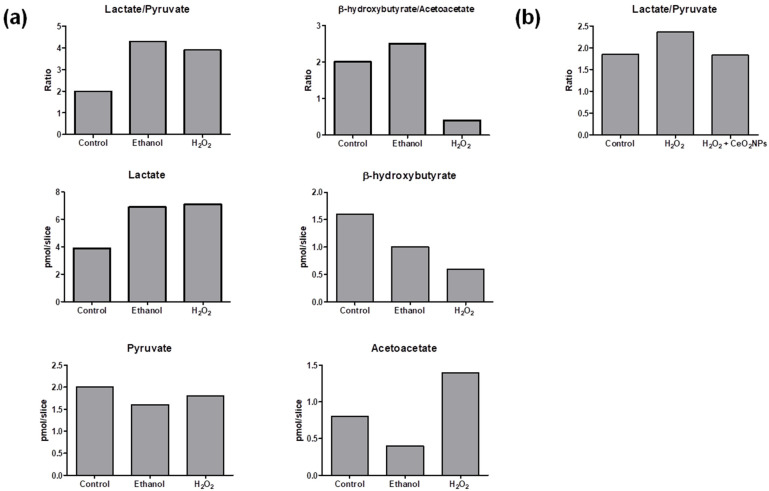
(**a**) Ratios of lactate/pyruvate and β-hydroxybutyrate/acetoacetate in precision-cut liver slices incubated under control conditions (control), exposed to ethanol (17 mM) or H_2_O_2_ (1.5 mM). (**b**) Ratios of lactate/pyruvate in human hepatic cells (HepG2) cultivated under normal conditions (control), stimulated with 2 mM H_2_O_2_ (H_2_O_2_) and stimulated with 2 mM H_2_O_2_ and treated with CeO_2_NPs (5 µg/mL) (H_2_O_2_ + CeO_2_NPs).

The method was applied to measure lactate/pyruvate ratio (as an indication of the cytosolic redox state) and β-hydroxybutyrate/acetoacetate ratio (as mitochondrial surrogate redox marker) in liver slices. Results are shown in Figure 3a. The presence of ethanol in the medium of incubated precision-cut liver rat slices resulted in an augmented lactate/pyruvate ratio in comparison to non-exposed control slices. This has been previously observed and attributed to the generation of cytosolic NADH through oxidation of ethanol via alcohol dehydrogenase [30]. A similar increase of lactate/pyruvate ratio was observed in precision-cut liver slices incubated with H_2_O_2_. Liver slices slightly augmented the β-hydroxybutyrate/acetoacetate ratio in response to ethanol, whereas H_2_O_2_ exposure strongly decreased the β-hydroxybutyrate/acetoacetate ratio suggesting a more marked effect of H_2_O_2_ in the mitochondrial redox state.

The method was also applied to evaluate cytosolic redox changes in human hepatic cells HepG2 cultured under three different experimental conditions. Although β-hydroxybutyrate and acetoacetate levels were below the limit of quantification, lactate and pyruvate quantification was achieved in these cells. The obtained lactate/pyruvate ratios are shown in Figure 3b. In agreement with results observed in precision-cut liver slices from rats, an increase of the lactate/pyruvate ratio was also observed in cells treated with H_2_O_2_ compared with the control group (cells incubated only with Dulbecco’s Modified Eagle Medium (DMEM)). In addition, cells incubated with H_2_O_2_ and exposed to CeO_2_NPs presented a reduction of lactate/pyruvate ratio in comparison with cells incubated with H_2_O_2_ alone, which is consistent with the well-known property of this nanomaterial to participate in redox reactions [31].

Although the described method in this study had an intended use of evaluating the cellular redox state by measuring the lactate/pyruvate ratio and β-hydroxybutyrate/acetoacetate ratios, the proposed method may also be suitable for other clinical or experimental situations, such as the evaluation of metabolic acidosis [8] or inherited metabolic diseases [9], where the quantification of one or more of these metabolites can be useful for the detection, diagnosis and treatment follow-up of specific conditions. Finally, and in contrast to other methods, the current method enables the simultaneous determination of the four metabolites and has been specifically validated both in plasma and in liver tissue in order to concomitantly assess the free NAD^+^/NADH state in cytosol and mitochondria.

## 3. Materials and Methods

### 3.1. Chemical Reagents

Sodium l-lactate, sodium pyruvate, sodium (R)-3-hydroxybutyrate, lithium acetoacetate, sodium l-lactate-3-^13^C, sodium pyruvate-1-^13^C, 5-sulfosalicylic acid hydrate, barium hydroxide, zinc sulfate, o-phenyelendiamine, BSTFA:TMCS (99:1), pyridine and ethyl acetate were obtained from Sigma-Aldrich (St. Louis, MO, USA)). β-Hydroxybutyrate-d_4_ was obtained from Cayman Chemical (Ann Arbor, MI, USA). Hydrochloric acid (HCl, 37%) was purchased from Panreac (Barcelona, Spain). Ultrapure water was obtained using a Millipore Milli-Q purification system (Synergy®, Merck Millipore, Burlington, MA, USA).

### 3.2. Instrumentation

GC-MS measurements were performed on a Shimadzu GCMS-QP2010 Ultra instrument (Kyoto, Japan). Splitless mode (valve opened at 1 min) was used to inject the final extracts into the gas-chromatograph interfaced with a mass selective detector. The chromatographic column was a Sapiens-5MS+ capillary column (30 m × 0.25 mm internal diameter × 0.25 μm film thickness) from Teknokroma (Barcelona, Spain). with helium as a carrier gas at a constant velocity (50 cm/s). The oven temperature conditions were as follows: started at 100 °C, maintained at this temperature for 2 min, elevated at 15 °C min^−1^ to 115 °C, increased at 80 °C min^−1^ until 300 °C and maintained for 6 min at 300 °C. The total run time was 11 min. The ion source and transfer line temperatures were set to 250 °C and 280 °C, respectively. Following a 2.5 min solvent delay, the mass detector was operated in synchronous selected ion monitoring (SIM) mode (*m*/*z* 117, 118, 217, 218, 191, 195, 231) using a dwell time of 150 ms and scan mode ranging from *m*/*z* 65 to *m*/*z* 280 *m*/*z* using a dwell time of 3 ms. Identification of the analytes in the sample extracts was achieved via GC retention time and comparison with reference standards. One μL was injected into the chromatographic system and four pre- and post-injection washes (in cyclohexane) were performed between injections.

### 3.3. Preparation of Stock Solutions, Working Solutions, Calibrators and Quality Controls Samples

Lactate, pyruvate, β-hydroxybutyrate and acetoacetate are endogenous compounds present in blank plasma and tissues. Therefore, calibration curves were prepared in water as free surrogate matrices. The analytical response differences between serum and free surrogate matrices were evaluated via a recovery assessment. Stock solutions were prepared by mixing and diluting the four metabolites in water to a final concentration of 20 mM for each metabolite and storing at −80 °C. Six-point calibration curves were prepared for the calibration of lactate (0.01, 0.025, 0.1, 0.25, 1 and 5 mM) and pyruvate and acetoacetate (0.001, 0.01, 0.025, 0.1, 0.25 and 1 mM) by diluting stock solutions in water. For β-hydroxybutyrate, a seven-point calibration curve was prepared (0.001, 0.01, 0.025, 0.1, 0.25, 1 and 5 mM).

For quality controls (QC), three concentrations were prepared for lactate (0.05, 0.5 and 2.5 mM) and for pyruvate and acetoacetate (0.005, 0.05 and 0.5 mM) and four concentrations for β-hydroxybutyrate (0.005, 0.05, 0.5 and 2.5 mM) by serially adding stock solutions in water.

The internal standards stock solutions of sodium l-lactate-3-^13^C, sodium pyruvate-1-^13^C and β-Hydroxybutyrate-d_4_ were prepared at a concentration of 10 mM in water and stored at −20 °C. A combined internal standard working solution was prepared at a final concentration of 6 mM of l-lactate-3-^13^C and 2 mM of sodium pyruvate-1-^13^C and β-Hydroxybutyrate-d_4_.

### 3.4. Sample Preparation

**Figure 4:**
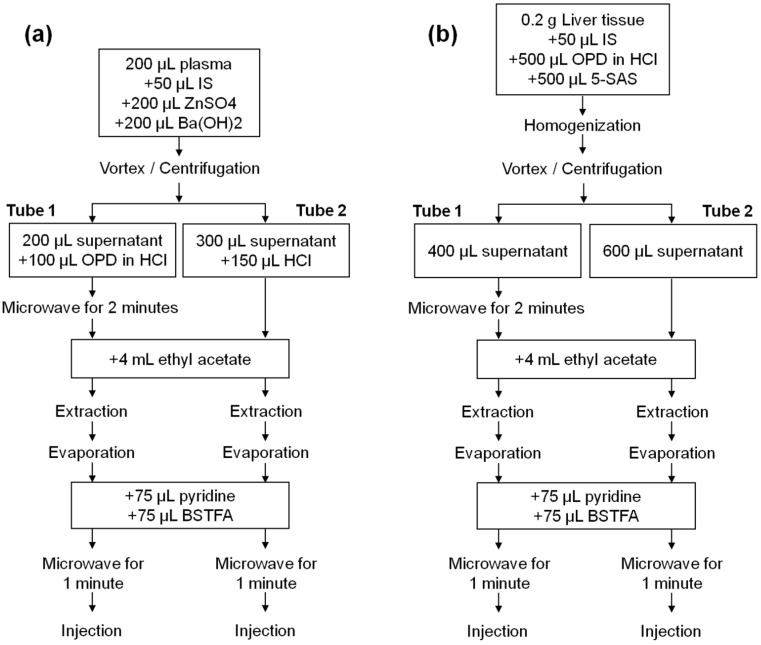
(**a**) Schema of the assay procedure for the determination of lactate and pyruvate (Tube 1) and β-hydroxybutyrate and acetoacetate (Tube 2) in plasma samples. (**b**) Schema of the assay procedure for determination of lactate and pyruvate (Tube 1) and β-hydroxybutyrate and acetoacetate (Tube 2) in liver samples. 5-SAS: 5-sulfosalicylic acid; BSTFA: bis(trimethylsilyl)trifluoroacetamide; HCl: hydrochloric acid; IS: internal standard; OPD: o-phenylenediamine.

Figure 4a shows a schema of the assay procedure for the determination of lactate, pyruvate, β-hydroxybutyrate and acetoacetate in plasma samples. First, 50 µL of internal standard working solution were added to 200 µL of sample. After adding 200 µL of ZnSO_4_ and 200 µL Ba(OH)_2_ for protein precipitation, samples were centrifuged and supernatant split in two tubes (tube 1 and tube 2). The extracts from tube 1 and tube 2 were handled and injected in parallel, resulting in two chromatograms for each initial sample. Tube 1 (lactate and pyruvate analysis) consisted of 200 µL of supernatant that was derivatized with 100 µL of o-phenylenediamine in 4 M HCl solution via microwave irradiation for 2 min. Tube 2 (β-hydroxybutyrate and acetoacetate analysis) consisted of 300 µL of supernatant that was acidified with 150 µL of 4 M HCl. Both tubes 1 and 2 were then extracted with 4 mL of ethyl acetate and evaporated to dryness under nitrogen at 37 °C after centrifugation. Trimethylsilyl ether derivatives (TMS) were formed via derivatization (75 μL BSTFA:TMCS 99:1 and 75 μL of pyridine, microwave irradiation for 1 min). Calibrators and QC samples were processed in the same manner but without protein precipitation. Figure 4b shows a schema of the assay procedure for the determination of lactate, pyruvate, β-hydroxybutyrate and acetoacetate in rat liver samples. Two hundred mg of liver were homogenized together with 0.5 mL 4% o-phenylenediamine (in 4 M HCl solution), 0.5 mL 16% sulfosalicylic acid and 50 µL of internal standard working solution. Samples were centrifuged and supernatant split in two tubes (tube 1 and tube 2). The extracts from tube 1 and tube 2 were handled and injected in parallel, resulting in two chromatograms for each initial sample. Tube 1 (lactate and pyruvate analysis) consisted of 400 µL of supernatant that was derivatized via microwave irradiation for 2 min. Tube 2 (β-hydroxybutyrate and acetoacetate analysis) consisted of 600 µL of supernatant. Both tube 1 and 2 were then extracted with 4 mL of ethyl acetate and evaporated to dryness under nitrogen at 37 °C after centrifugation. Trimethylsilyl ether derivatives were formed via derivatization (75 μL BSTFA:TMCS 99:1 and 75 μL of pyridine, microwave irradiation for 1 min).

### 3.5. Microwave-Assisted Derivatization

Derivatization yields obtained when performing the quinoxalinol derivative of pyruvate under thermal or microwave-assisted energy transfer were compared. When conducting the thermal block heating approach, quinoxalinol derivatives were formed by adding 200 μL of 4% o-phenylenediamine (in 4 M HCl solution) to the extracts and heating for 15, 30 or 45 min at 90 °C. MAD samples were similarly prepared and irradiated on a domestic microwave for 1.0, 1.5 or 2 min. After ethyl acetate extraction and evaporation, O-TMS-quinoxalinol derivatives of pyruvate were formed by adding 75 μL BSTFA:TMCS 99:1 and 75 μL of pyridine that were microwave irradiated for 1 min. The relative response factors (RRFs) for pyruvate were calculated by comparing the area ratios obtained via MAD and thermal derivatization. Specifically, the obtained area of each metabolite was divided by the mean area yielded via thermal block derivatization for 45 min.

### 3.6. Method Validation

#### 3.6.1. Linearity of Calibration Curves and Matrix Effect

Linearity was evaluated over a range between 0.01 and 5 mM (lactate), 0.001 and 1 mM (pyruvate and acetoacetate) and 0.001 and 5 mM (β-hydroxybutyrate) using six (lactate, pyruvate and acetoacetate) or seven (β-hydroxybutyrate) concentrations. Complete calibration curves were analyzed on 3 separate days. A weighted linear (β-hydroxybutyrate) or weighted quadratic regression (lactate, pyruvate and acetoacetate) was used to plot the peak area ratio (metabolite relative to internal standard) versus the corresponding concentration. An isotopic internal standard was used for each metabolite except for acetoacetate for which β-Hydroxybutyrate-d_4_ was used as internal standard. Slope, y-intercept and correlation coefficient were calculated for each standard curve. A minimum value of r^2^ = 0.99 was required to pass this validation step. Precision and accuracy versus nominal concentration of standards were also calculated.

The analytical responses of the four metabolites in plasma and liver were assessed to ensure that the calibration curve built in water standards and without previous protein precipitation could be used to quantify plasma and liver samples. The slope coefficient (α) of 3-point QC regression lines spiked in human plasma or rat liver homogenates from two different sources were compared with their respective regression lines spiked in water.

#### 3.6.2. Accuracy and Precision

Evaluation of the accuracy and precision of the analysis method was based on back-calculated results of multiple measurements of three QC, the lower limit of quantification (LloQ) and the upper limit of quantification (UloQ). The lowest and highest calibration values were used as LloQ and UloQ, respectively. Additionally, precision of the assay was evaluated in both human plasma and rat liver homogenates. Concentration results were measured using complete external standard curves in all cases. Accuracy and precision were evaluated on three replicates performed for each concentration on the same day (intra-day) and on three different days (inter-day). Accuracy was assessed as the difference between calculated concentrations of the metabolites with theoretical concentrations expressed in percent and should be within the 100 ± 15% limits. Precision at each concentration was expressed as relative standard deviation (%RSD) for each QC and should not exceed 15%. In the case of the LloQ, accuracy should be within 100 ± 20% and precision below 20%.

#### 3.6.3. Recovery, Selectivity, Carry-Over and Stability of Derivatives

The recovery of added lactate, pyruvate, β-hydroxybutyrate and acetoacetate was determined by comparing the expected and obtained concentrations in plasma and liver samples used in the matrix effect evaluation with the formula Recovery (%) = (C_observed_ − C_expected_)/C_expected_. Concentration results were measured using complete external standard curves in all cases. The selectivity was investigated by analyzing 10 different human plasma sources and 3 different rat liver sources and was indicated by the absence of any endogenous interference at retention times of the metabolites. The carry-over was evaluated by injecting 1 µL of cyclohexane immediately after the injection of a standard with UloQ concentration (5 mM) on three separate occasions. The stability of MAD extracts on the autosampler was evaluated by reanalyzing QC samples stored inside the autosampler at ambient temperature for up to 96 h.

### 3.7. Method Application

To test the applicability of the method, metabolic ratios were measured in precision-cut liver slices from rats and were also quantified in cultured human hepatic cells (HepG2 cells) under different experimental conditions challenging the redox state.

#### 3.7.1. Precision-Cut Liver Slices from Rats

A 350 g female Wistar rat (Charles-River, Saint Aubin les Elseuf, France) was housed under controlled conditions with a 12 h light/dark cycle and fed ad libitum with standard chow. On the day of the experiment, the rat was sacrificed with an isofluorane overdose (Forane, Abbott Laboratories S.A., Madrid, Spain). The study was performed in agreement with the criteria of the Investigation and Ethics Committee of the Hospital Clinic (Barcelona, Spain). The animal protocol used in this work was evaluated and approved by the Committee of Animal Experimentation (CEEA) of the University of Barcelona (Register 357/19, 24 July 2020) The liver was excised and placed into ice-cold Storage Solution Bel-Gen (IGL, Lissieu, France). Five mm diameter core biopsies were obtained and embedded in UltraPure low melting point agarose (3% (*w*/*v*), Invitrogen, Bleiswijk, The Netherlands) prewarmed at 37 °C. Once agarose solidified, 250 µm thick precision-cut liver slices were prepared using Leica VT1200 S vibratome (Leica Microsystems, Nussloch, Germany) filled with ice-cold Krebs-Henseleit buffer which consisted of 25 nM NaHCO_3_ (Sigma-Aldrich, Saint Louis, MO, USA), 25 mM d-glucose (Sigma-Aldrich, St. Louis, MO, USA). ) and 10 mM HEPES (Sigma-Aldrich), previously saturated with carbogen and adjusted to pH 7.4. Slices were preincubated for 1 h in 12-well plates containing 1.3 mL of prewarmed and preoxygenated Williams Medium E (Gibco, Paisley, UK) supplemented with 50 µg/mL gentamycin (Gibco) and 25 mM d-glucose (Sigma-Aldrich) in a humidified atmosphere of 40% O₂/ 5% CO₂ at 37 °C while gently shaken at 90 revolutions per minute. Thereafter, slices were transferred for 1 h to new prewarmed and preoxygenated medium, medium containing 17 mM ethanol or medium containing 1.5 mM H_2_O_2_. At the end of the experiment, duplicate slices for each condition of approximately 5 mg each were snap-frozen for metabolite measurement as previously described and depicted in Figure 4b.

#### 3.7.2. Cultured Human Hepatic Cells

HepG2 cells were obtained from American Type Culture Collection (ATCC, Manassas, VA, USA). Four 100 mm dishes were seeded with 3 × 10^6^ cells each. Cells were grown to confluence in Dulbecco’s modified Eagle medium (DMEM), supplemented with 10% fetal bovine serum (FBS), 50 U/mL penicillin and 50 μg/mL streptomycin, in a humidified atmosphere of 5% CO_2_ at 37 °C. After 24 h, the medium was replaced with a serum-free DMEM overnight. Then, this medium was removed and each plate was incubated for one hour with a different experimental condition: DMEM (control group), DMEM containing 2 mM H_2_O_2_ (H_2_O_2_ group) or DMEM containing 2 mM H_2_O_2_ and 5 µg/mL of cerium oxide nanoparticles (H_2_O_2_ + CeO_2_NPs group). After one hour of incubation, cells were lysed and duplicate measurements of lactate and pyruvate were performed as showed in Figure 4b. Cerium oxide nanoparticles (4 nm) were synthesized and characterized as previously described [32].

### 3.8. Statistical Calculations

Statistical calculations were performed with R statistical software version 4.0.2 (The R Foundation for Statistical Computing, Vienna, Austria) and the GraphPad Prism 8 (GraphPad Prism Software Inc., San Diego, CA, USA).

## 4. Conclusions

A sensitive GC-MS method was developed and validated for the quantitative measurement of lactate/pyruvate and β-hydroxybutyrate/acetoacetate in human plasma and rat liver tissue with acceptable accuracy and imprecision over a wide range of concentrations. A simple liquid-liquid extraction method was used and derivatization was reduced to a few minutes of microwave irradiation. The method may be useful for the quantification of in vivo redox states based on free NAD+/NADH in biological samples.

## Figures and Tables

**Table 1:** Selected ion monitoring values (*m*/*z*) and retention time of metabolites. TMS: trimethylsilyl.

Metabolite	Derivative	Selected Ion (*m*/*z*)	Retention Time (min)
Lactate	2-TMS	117	2.9
l-lactate-3-13C	2-TMS	118	2.9
Pyruvate	TMS-quinoxalinol	217	5.1
Pyruvate-1-13C	TMS-quinoxalinol	218	5.1
β-hydroxybutyrate	2-TMS	191	3.7
β-Hydroxybutyrate-d4	2-TMS	195	3.7
Acetoacetate	2-TMS	231	4.2

**Table 2:** Precision and accuracy values of the calibration curve standards (*n* = 3). A: accuracy; P: precision; Std: standard.

		Lactate		Pyruvate		β-Hydroxybutyrate		Acetoacetate	
Standard	mM	A (%)	P (%)	A (%)	P (%)	A (%)	P (%)	A (%)	P (%)
Std 1	0.001	-	-	93.3	12.4	100.0	0.0	120.0	22.0
Std 2	0.01	101.7	14.7	110.3	4.6	99.7	6.0	86.7	15.7
Std 3	0.025	98.9	12.9	100.9	9.9	98.5	8.5	92.1	7.5
Std 4	0.1	99.4	6.3	101.7	9.2	95.7	1.8	97.0	9.4
Std 5	0.25	105.3	8.0	99.8	6.2	92.7	11.1	102.8	5.6
Std 6	1	98.4	3.5	100.0	0.6	93.9	10.0	99.7	0.5
Std 7	5	99.6	1.4	-	-	101.8	1.4	-	-

**Table 3:** Recovery of added metabolites in human plasma and rat liver samples.

		Human Plasma			Rat Liver		
	Added (mM)	Detected (mM)	Expected (mM)	Recovery (%)	Detected (mM)	Expected (mM)	Recovery (%)
Lactate	-	0.37	-	-	0.66		
	0.1	0.41	0.47	87.2	0.69	0.76	90.8
	0.4	0.79	0.77	102.6	1.10	1.06	103.8
Pyruvate	-	0.13	-	-	0.13		
	0.1	0.22	0.23	95.7	0.23	0.23	100.0
	0.4	0.53	0.53	100.0	0.57	0.53	107.5
β-hydroxybutyrate	-	0.12	-	-	0.17		
	0.1	0.20	0.22	90.0	0.24	0.22	88.9
	0.4	0.53	0.52	101.9	0.53	0.52	98.2
Acetoacetate	-	0.07	-	-	0.07		
	0.1	0.16	0.17	94.1	0.15	0.17	88.2
	0.4	0.53	0.47	112.8	0.41	0.47	87.2

**Table 4:** Intra-day and inter-day precision expressed as relative standard deviation and accuracy values of quality. A: accuracy; P: precision; QC: quality controls.

		Intra-Day (*n* = 3)		Inter-Day (*n* = 3)	
	mM	A (%)	P (%)	A (%)	P (%)
Lactate					
QC2	0.05	106.3	5.0	98.5	15.3
QC3	0.5	101.2	9.1	105.1	8.4
QC4	2.5	-	-	95.9	3.0
Pyruvate					
QC1	0.005	-	-	102.7	14.1
QC2	0.05	90.8	10.3	103.1	6.4
QC3	0.5	91.4	8.5	101.6	8.2
β-hydroxybutyrate					
QC1	0.005	-	-	91.3	6.7
QC2	0.05	90.3	11.5	96.4	10.9
QC3	0.5	93.8	11.2	91.0	9.6
QC4	2.5	-	-	101.3	11.9
Acetoacetate					
QC1	0.005	-	-	86.7	15.0
QC2	0.05	87.7	12.2	95.5	8.6
QC3	0.5	85.0	13.8	109.0	9.0

**Table 5:** Intra-day and inter-day precision expressed as relative standard deviation of plasma and liver samples. P: precision.

	Intra-Day (*n* = 3)		Inter-Day (*n* = 3)	
	Mean (mM)	P (%)	Mean (mM)	P (%)
Lactate				
Plasma	1.3875	6.5	1.8025	6.2
Liver	0.9253	1.8	1.3778	10.1
Pyruvate				
Plasma	0.1319	7.9	0.1459	8.9
Liver	0.0502	14.9	0.1984	7.2
β-hydroxybutyrate				
Plasma	0.0605	4.4	0.0663	8.6
Liver	0.0752	4.6	0.5991	10.1
Acetoacetate				
Plasma	0.0107	2.9	0.0115	10.5
Liver	0.0047	6.2	0.0310	21.9

**Table 6:** Stability in the autosampler at ambient temperature expressed as accuracy values of quality control samples after 24 h and 96 h. QC: quality control.

	Mean (mM)	Accuracy (%)		Precision (%)
		24 h	96 h	
Lactate				
QC2	0.05	95.4	90.3	5.1
QC3	0.5	95.7	96.1	2.4
QC4	2.5	94.0	102.0	4.2
Pyruvate				
QC1	0.005	108.1	114.5	6.8
QC2	0.05	95.5	106.8	5.7
QC3	0.5	101.6	110.8	5.6
β-hydroxybutyrate				
QC1	0.005	100.0	98.0	1.2
QC2	0.05	96.8	97.3	1.8
QC3	0.5	102.4	104.4	2.1
QC4	2.5	86.0	86.4	8.8
Acetoacetate				
QC1	0.005	100.0	106.1	3.4
QC2	0.05	93.0	104.5	5.8
QC3	0.5	96.2	98.7	2.0

## Data Availability

Not applicable.

## Supplementary Materials

The following are available online at https://www.mdpi.com/article/10.3390/ijms22094752/s1, Figure S1: Mass spectra of lactate-2TMS, pyruvate-TMS-quinoxalinol, β-hydroxybutyrate-2TMS and acetoacetate-2TMS, Table S1: Mean slope values of regression lines obtained from three-point spiked human serums and rat livers compared with the respective slope values of regression lines obtained from three-point spiked water samples (n = 2).
