# Supplementary material for: Validation of a Gas Chromatography-Mass Spectrometry Method for the Measurement of the Redox State Metabolic Ratios Lactate/Pyruvate and β-Hydroxybutyrate/Acetoacetate in Biological Samples
Source: Int J Mol Sci. 2021 Apr 30;22(9):4752. doi: 10.3390/ijms22094752 (PMC8125771; doi:10.3390/ijms22094752)

Validation of a gas chromatography-mass spectrometry method for the measurement of the redox state metabolic ratios lactate/pyruvate and β-hydroxybutyrate/acetoacetate in biological samples

Robin Wijngaard [1], Meritxell Perramón [1], Marina Parra-Robert [1], Susana Hidalgo [1], Gina Butrico [2], Manuel Morales-Ruiz [1,3,4], Muling Zeng [5], Eudald Casals [5], Wladimiro Jiménez [1,3], Guillermo Fernández-Varo [1,3,*], Gerald I Shulman [2], Gary W Cline [2], and Gregori Casals [1,4,*]

[1] Service of Biochemistry and Molecular Genetics, Hospital Clinic Universitari, Centro de Investigación Biomédica en Red de Enfermedades Hepáticas y Digestivas (CIBERehd), Institut d'Investigacions Biomèdiques August Pi i Sunyer (IDIBAPS), Carrer de Villarroel, 170, 08036 Barcelona, Spain; wijngaard@clinic.cat (R.W.); mperramon@clinic.cat (M.P.); mparra@clinic.cat (M.P.-R.); shidalg1@clinic.cat (S.H.); morales@clinic.cat (M.M.-R.); wjimenez@clinic.cat (W.J.); guillermo.fernandez@ciberehd.org (G.F.-V.); casals@clinic.cat (G.C.)

[2] Department of Internal Medicine, Yale School of Medicine, New Haven, CT; ginabutrico@gmail.com (G.B.); gerald.shulman@yale.edu (G.S.); gary.cline@yale.edu (G.W.)

[3] Departament of Biomedicine, University of Barcelona, 08036 Barcelona, Spain

[4] Working group for the biochemical assessment of hepatic disease-SEQCML, 08036 Barcelona, Spain

[5] School of Biotechnology and Health Sciences, Wuyi University, 99 Yingbing Middle Rd., Jiangmen, 529020, China; mulingzeng@163.com (M.Z.); eudaldcm@gmail.com (E.C.)

* Correspondence: guillermo.fernandez@ciberehd.org (G.F.-V.); casals@clinic.cat (G.C.) Tel.: (optional; include country code; if there are multiple corresponding authors, add author initials)

Keywords: redox state; GC-MS; microwave-assisted derivatization; nicotinamide adenine dinucleotide; ketone bodies

Table S1. Mean slope values of regression lines obtained from three-point spiked human serums and rat livers compared with the respective slope values of regression lines obtained from three-point spiked water samples (n=2).

	Lactate	Pyruvate	β-hydroxybutyrate	Acetoacetate
Water	1.63	1.84	3.00	5.47
Plasma	1.59	1.89	3.15	4.32
Liver	1.54	1.84	3.02	4.49

Figure S1. Mass spectra of lactate-2TMS **(a)**, pyruvate-TMS-quinoxalinol **(b)**, β-hydroxybutyrate-2TMS **(c)** and acetoacetate-2TMS **(d)**.

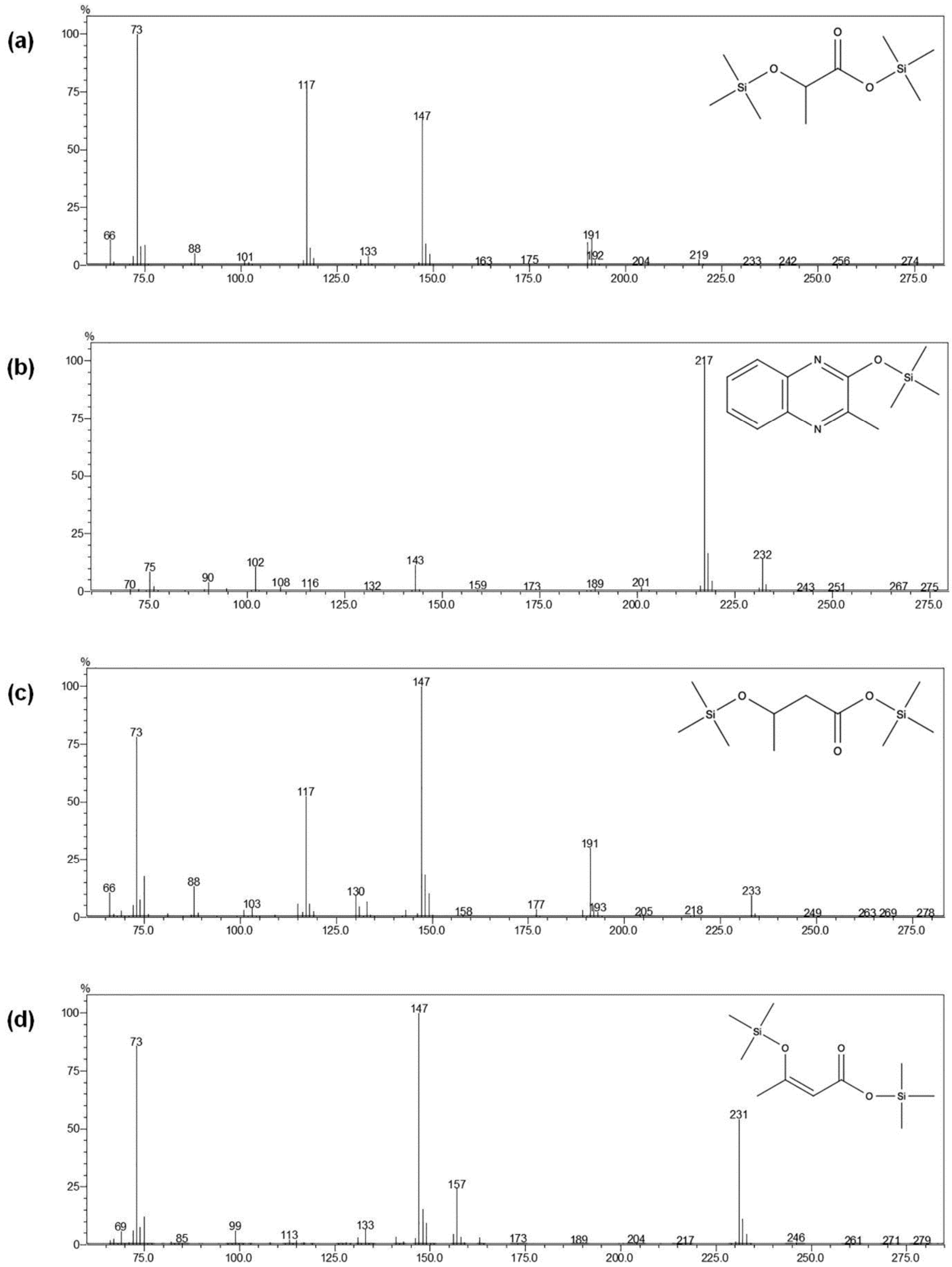

Supplement: Supplementary file 1 [file ijms-22-04752-s001.zip › ijms-1170133-supplementary.pdf]
